# Skeletal Muscle Denervation: Past, Present and Future

**DOI:** 10.3390/ijms23147489

**Published:** 2022-07-06

**Authors:** Tatiana Y. Kostrominova

**Affiliations:** Department of Anatomy, Cell Biology and Physiology, Indiana University School of Medicine, Northwest, Gary, IN 46408, USA; tkostrom@iun.edu

This Special Issue presents some of the most recent studies on the skeletal muscle denervation. Skeletal muscle innervation comprises stable and functional nerve interactions with muscles via neuromuscular junctions (NMJ). It is critical for the maintenance of normal muscle structure and function [1]. Loss of motor innervation induces rapid skeletal muscle fiber degeneration with the activation of atrophy-related signaling and subsequent disassembly of the sarcomeres [2]. This results in a loss of muscle function. Some of these changes can be observed within just a few hours after denervation [3].

Denervation can be caused by numerous abnormalities: nerve injury due to trauma, nerve degeneration due to the loss of myelination, destruction of the NMJ due to the autoimmune disorders or aging-related loss of motor neurons and many other disorders [1]. 

Denervation-induced degradation processes in skeletal muscle are relatively well described [4]. The interest in the denervation research is increasing with time. The first publications found in PubMed are dated to 1915, while in 2021, there were 433 publications on denervation. It is well established that one of the most critical degradation pathways in skeletal muscle is ubiquitin/E3 ligases [5]. Signaling pathways that activate calpains are important for the degradation of skeletal muscle structural (sarcomeric) proteins [6]. Muscle and bone function as one musculoskeletal unit [7]. When there is functional and structural damage to the skeletal muscle, this leads to bone abnormalities and vice versa. The current Special Issue includes a review by Dr. Klein describing role of transforming growth factor-beta (TGF-beta) in skeletal muscle wasting [8]. Bone remodeling/resorption releases TGF-beta, which promotes muscle fibrosis and atrophy. Considering that severe trauma causes substantial damage to bone as well as muscle fibers, large quantities of TGF-beta released during bone regeneration/remodeling could potentially have negative impact on post-trauma muscle recovery.

It is also well established that, along with degradation processes, there is a simultaneous activation of compensatory processes in denervated muscle. Muscle collagen breakdown is inhibited after denervation, leading to collagen accumulation [9]. The activation of muscle stem cells (satellite cells) during prolonged denervation helps to maintain muscle fiber integrity [10]. These processes allow the survival of small atrophic skeletal muscle fibers, even after a very long period of denervation [11]. Strategies that decrease the degeneration of denervated muscle and increase the compensatory activation of protective processes can be beneficial in muscle recovery during re-innervation.

With repaired innervation and proper re-establishment of the NMJ, the atrophic muscle fibers can be restored to their original size (Figure 1). This process is much more successful in young humans and animals, and it diminishes with aging [12]. Several studies have tried to address the differences in the re-innervation processes in young versus old individuals in order to develop better treatment strategies for the elderly. The current Special Issue features a review by Dr. Iyer and colleagues on differences in NMJ between young and old individuals and the role of these changes in age-associated skeletal muscle abnormalities and in neuromuscular disease [1]. Since NMJ plays a key role in musculoskeletal impairment with aging and in many human disorders, the authors provide an overview of potential therapeutic targets in neuromuscular injury and disease [1]. A research paper in this Special Issue describes the role of neuronal CuZnSOD deletion on muscle mitochondria and calcium handling [13]. The disruption of neuronal redox status leads to mitochondrial dysfunction that contributes to NMJ deterioration and to sarcopenia.

Denervation not only leads to muscle atrophy but also affects muscle metabolic functions. The study from Dr. Witczak’s laboratory published in the current Special Issue describes the effect of denervation on insulin resistance [14]. Although both slow (soleus) and fast (extensor digitorum longus or EDL) muscle atrophied equally to about 40% after 28 days of denervation, changes in insulin sensitivity were completely different. GLUT 4 transporter was increased in fast EDL muscle and decreased in slow soleus muscle. This correlated with the development of insulin resistance in soleus but increased insulin sensitivity in the EDL muscle. Considering that aging-associated denervation of skeletal muscle predominantly affects fast muscle fibers with relative sparing of slow muscle fibers [1], it is interesting what effect this could have on glucose metabolism in the elderly.

While we know a lot about processes that regulate skeletal muscle atrophy after denervation, we still do not have optimized treatment for the prevention of skeletal muscle atrophy after denervation or for the improved reinnervation. This is a very active area of research, and many laboratories are trying to find answers to these questions. Electrical stimulation of the denervated muscle shows good results for diminishing denervation-induced muscle atrophy [15]. While it allows for maintaining muscle fiber size, it does not improve re-innervation after prolonged denervation [16].

One of the innovative approaches to reducing denervation-induced muscle atrophy and preserving muscle function is described in the original study presented in the current Special Issue [17]. Cervical spinal cord injury (SCI) promotes muscle atrophy and weakness of the diaphragm. Hyperbaric oxygen exposure for one hour during ten consecutive days post-injury improved diaphragm-specific force production, attenuated muscle fiber atrophy, mitochondrial dysfunction and ROS production in rats [17]. Hyperbaric oxygen treatment increases the level of antioxidants, which mitigate the action of reactive oxygen species released after the denervation of the diaphragm muscle. For example, after SCI, glutathione peroxidase mRNA expression was increased in the hyperbaric-oxygen-treated group more than sixfold and protein expression more than twofold when compared with innervated control and with the SCI group without oxygen treatment. SCI increased the expression of E3 ubiquitin ligases MAFbx and MuRF1 in the diaphragm. Remarkably, the hyperbaric oxygen treatment completely prevented this increase in the diaphragm muscle. The findings presented in this study suggest that a similar approach can be potentially used in humans to alleviate cervical-spinal-cord-injury-induced diaphragm muscle atrophy.

Some of the approaches used in the attenuation of muscle fiber degeneration after denervation include the use of natural compounds. This topic is reviewed in the current Special Issue by Shirakawa and colleagues [18]. The review focuses on maxillofacial muscle atrophy and differences between facial and trunk muscles. One of the reviewed compounds that might be beneficial for the prevention of muscle atrophy is royal jelly produced by honeybees. Another reviewed compound is geranylgeraniol, a C20 isoprenoid that naturally occurs in several foods. Oral treatment of rats with royal jelly or with geranylgeraniol had beneficial effects on denervated muscle and promoted myogenesis of myoblasts in vitro. Other reviewed natural compounds with beneficial effects on muscle include soy proteins, isoflavones, Vitamins (C, D and E) and capsaicin. Along with other available treatments, natural compounds can be potentially used in humans as a safe adjuvant therapy to diminish skeletal muscle atrophy.

Although considerable advances were achieved in recent years in our understanding of muscle denervation/re-innervation processes, the clinical application of this knowledge requires significant improvement and optimization. This should include a complex approach that involves the engagement of all critical pathways and approaches leading to the successful maintenance and repair of skeletal muscle motor innervation.

## Figures and Tables

**Figure 1:**
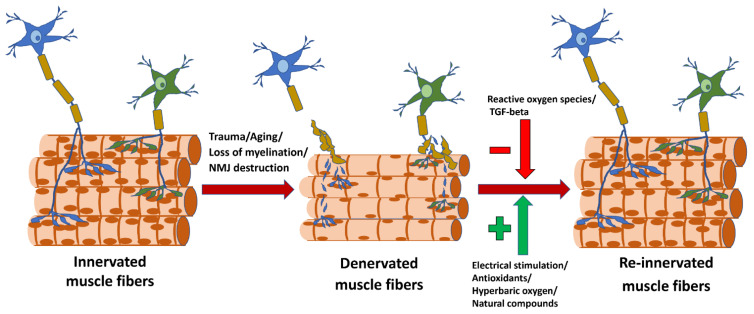
Schematic picture of muscle innervation/denervation/re-innervation processes.
